# Echocardiographic and electrocardiographic assessments in patients with psoriasis

**DOI:** 10.22088/cjim.12.2.162

**Published:** 2021-03

**Authors:** Zeinab Aryanian, Iraj Jafaripour, Edris Kohneshin, Roghayeh Pourkia, Mohammad Taghi Hedayati Goudarzi, Soudabeh Tirgar Tabari, Azar Shirzadian Kebria, Farbod Zahedi Tajrishi, Mohammad Mostafa Ansari Ramandi

**Affiliations:** 1 Autoimmune Bullous Diseases Research Center, Tehran University of Medical Sciences, Tehran, Iran; 2 Department of Dermatology, Babol University of Medical Sciences, Babol, Iran; 3 Department of Cardiology, Babol University of Medical Sciences, Babol, Iran; 4 Student Research Committee, Babol University of Medical Sciences, Babol, Iran; 5 Cardiovascular Diseases Research Center, Birjand University of Medical Sciences, Birjand, Iran

**Keywords:** Psoriasis, P wave dispersion, Diastolic function, Systolic function, Pulmonary artery pressure

## Abstract

**Background:**

Psoriasis is a chronic inflammatory disease affecting many organs. Recent studies have demonstrated that psoriasis is associated with cardiovascular disorders. We investigated the echocardiographic and conduction system changes in psoriasis patients.

**Methods:**

In this case-control study, 36 psoriatic patients and 36 healthy controls were enrolled. Demographic and clinical data, echocardiographic and P wave dispersion (PWD) in 12-lead electrocardiogram were evaluated in both groups. We recruited patients with confirmed diagnosis via biopsy and have not been under recent systemic treatment. Patients with underlying cardiovascular disease were excluded from the study.

**Results:**

Mean age was 41.56±16.20 and 39.67±13.85 year in case and control groups, respectively. There was no significant difference in the baseline characteristics of the two groups. PWD was significantly higher in the case group (p<0.05). High pulmonary artery pressure was observed in 14 psoriatic patients and 1 individual in the control group (p<0.001). Left ventricular diastolic dysfunction was significantly higher among individuals who were above 60 years of age (p<0.01) but not significantly different between the two groups.

**Conclusion:**

Psoriatic patients are more susceptible to future development of atrial fibrillation because of higher PWD. There is no significant difference between the diastolic function in these patients.

Psoriasis is a chronic inflammatory skin disorder characterized by grey scaly plaques on the extensor surfaces of the body, scalp, navel and waist area (1, 2). Despite an estimated prevalence of 0.09 to 11.4% which varies from country to country, the etiology of the condition is not yet fully understood. Nevertheless, certain medications and infections as well as genetic factors, trauma, smoking, alcohol consumption, emotional stress and limited sunlight exposure are all known as triggering and/or exacerbating factors of the disorder (3-5). Studies have also revealed the involvement of several inflammatory markers including multiple cytokines produced by T-helper cells in the pathogenesis of psoriasis (6). The inflammation and oxidative stress produced in the course of psoriasis contributes to a higher risk of developing progressive diseases such as metabolic syndromes and coronary artery disease as well as an increased risk of mortality followed by myocardial infarction (7-9). The inflammatory state seen in disorders such as psoriasis are also associated with increasing the risk of developing cardiac arrhythmias such as atrial fibrillation (AF), which is the most common arrhythmia and associated with further increase in major adverse cardiovascular events (10-12).

P-wave dispersion (PWD), is an electrocardiographic marker defined as the difference between the highest (P_max_) and the lowest (P_min_) P wave duration in a standard 12-lead electrocardiogram. Evidence suggests that increased P-wave duration and dispersion increase the risk for developing AF (12). In a study done by Simsek et al., they showed that PWD is greater in patients having psoriasis (9). A study has shown patients with psoriasis having more diastolic dysfunction and higher end-diastolic and systolic diameters (1). However, in another study, there was no significant difference in the diastolic functions (13). Another study has also demonstrated lower systolic function in patients suffering from psoriasis (14). Because of the relatively high prevalence of psoriasis and the growing body of evidence pointing towards its potential effects on the cardiac conduction system and heart function, we performed this investigation to assess the electrocardiographic and echocardiographic alterations in psoriatic patients.

## Methods

**Study design: **We performed this case-control study on subjects who referred to Teaching hospital wards and clinics of Babol University of Medical Sciences in 2016 and 2017. Ethics committee approval was obtained from the institutional review board (Ethics code: MUBABOL.HRI.REC.1396.3). The sample size was estimated by previous study on PWD. Confidence level was 95% and the power was 80% with a standard deviation of 7.5 units. The calculated sample size for each group for finding a 5-unit difference in PWD was 36 individuals. The formula was as follows: 

$N=\frac{{\left(7.52+7.5\right)}^{2}{\left(1.96+0.84\right)}^{2}}{5^{2}}=36$

Thirty-six psoriatic patients and 36 healthy controls were enrolled in the study as case and control groups, respectively. Initial skin biopsies were taken from each participant to confirm or rule out psoriasis. 

**Inclusion and exclusion criteria: **The inclusion criteria for the patient group were a diagnosis of psoriasis confirmed via biopsy and having at least 20 years of age. Patients were otherwise healthy, without any underlying cardiovascular disorders. Pregnant women, patients with a recent history of systemic therapy or phototherapy and those with confirmed inflammatory, infectious and acute/chronic autoimmune diseases, heart failure, hepatic failure, malignancies and history of arrhythmias, atrial fibrillation, coronary artery disease, hypertension, diabetes mellitus and thyroid disorders were all excluded from the study. Patients using anti-arrhythmic, anti-histamine and anti-psychotic drugs were excluded as well. 

The effect of confounding factors such as the patients’ socio-economic status and other underlying factors was minimized by matching them between the two groups. Both groups were also matched in terms of age and sex and written informed consent was obtained from each subject before participation.

**Measurements: **After obtaining and recording the patients’ demographic, clinical and individual data, they were referred to Babol Shahid Beheshti Hospital, where they underwent 12-lead electrocardiography performed by an expert ECG operator. Manual measurement with hand-held calipers was performed by increasing the ECG rate to 50 mm/s and the voltage to 1 mV/cm, accompanied by use of 10 times magnification. Immediately afterwards, a board-certified cardiologist who was blinded to the study performed echocardiography for each patient in a left lateral decubitus position using a GE Healthcare Vivid S5 cardiovascular ultrasound device.

P-wave alterations as well as its dispersion in the ECG and echocardiographic markers (left ventricular ejection fraction, left ventricular diastolic function, left ventricular hypertrophy, valvular defects and pulmonary artery pressure) were all carefully observed and evaluated. 

**Statistical analysis: **The data were then statistically analyzed using Version 23 of SPSS software. T-test and chi-square tests were used to assess qualitative and quantitative data, respectively. Logistic regression was used to modify the confounding effect and a p-value of <0.05 was considered as statistically significant. We also divided the patients according to Psoriasis Area Severity Index (PASI) into three ranges: <7 (low), 7-12 (moderate) and >12 (high).

## Results

The duration between diagnosis and treatment of psoriatic patients ranged from 2 to 480 months (mean= 120.33±96 months). Mean age of participants was 41.56±16.20 and 39.67±13.85 years in case and control groups, respectively (P=0.597). Mean Body mass index (BMI) was 28.06±5.62 kg/m2 in the patient group and 26.67±4.00 kg/m2 in the control group. Table 1 demonstrates the demographic data of both study groups in detail.

Table 2 represents the clinical data as well as echocardiographic and P-wave information of the participants. There was no significant difference between case and control groups in terms of systolic and diastolic blood pressures, mean arterial pressure, heart rate and left ventricular ejection fraction (p=0.370, 0.384, 0.933, 0.991 and 0.219, respectively- table 2). On the other hand, P_max_ was higher in the control group (P=0.038) and P_min_ was lower in the case group (p<0.001). PWD was significantly higher in the case group compared with the control group (P=0.022). The prevalence of valvular abnormalities such as mitral regurgitation, tricuspid regurgitation, aortic regurgitation and aortic stenosis was the same between the two study groups (p>0.05). Left ventricular diastolic function was evaluated in both groups based on Doppler and tissue Doppler imaging, which yielded no significant difference between case and control groups (table 2). Abnormal pulmonary artery pressure (PAP) (>30 mmHg) was more prevalent in the case group (p<0.001) (table 2). Based on PASI classification, 28 patients had a low PASI, 7 had moderate and 1 had a high PASI. We found no association between PASI and the electrocardiographic or echocardiographic changes (table 3).

**Table 1:** Demographic characteristics of the study groups

**Variable**		**Case (Percentage)**	**Control (Percentage)**	**P value**
Age range (years)	≤39	15 (41.7%)	18 (50%)	0.713
	40-59	16 (44.4%)	15 (41.7%)	
	≥60	5 (13.9%)	3 (8.3%)	
Gender	Male	13 (44.8%)	16 (55.2%)	0.631
	Female	23 (53.5%)	20 (46.5%)	
BMI	18.5-24.9	11 (47.8%)	12 (52.2%)	0.834
	25-29.9	12 (46.2%)	14 (53.8%)	
	≥30	13 (56.5%)	10 (43.5%)	

BMI= Body Mass Index

**Table 2:** Clinical, echocardiographic and P wave data of case and control groups

**Variable**		**Case (Mean±SD or in percentage)**	**Control (Mean±SD or in percentage)**	**P value**
Systolic BP (mmHg)		115.14±14.71	112.36±11.18	0.370
Diastolic BP (mmHg)		69.86±8.15	71.53±8.00	0.384
Mean arterial pressure (mmHg)		84.95±9.88	85.14±8.62	0.933
Heart rate (bpm)		75.72±9.92	75.70±10.03	0.991
LVEF (%)		61.53±4.11	60.42±3.46	0.219
P_max_ (msec)		78.67±13.89	80.06±15.75	0.038
P_min_ (msec)		38.22±6.86	50.67±10.94	<0.001
PWD (msec)		40.72±10.84	35.11±9.43	0.022
Diastolic dysfunction	No	20 (43.5%)	26 (56.5%)	0.361
	Grade I	9 (64.3%)	5 (35.7%)	
	Grade II	7 (58.3%)	5 (41.7%)	
PAP	Normal	22 (38.6%)	35 (61.4%)	<0.001
	Abnormal	14 (93.3%)	1 (6.7%)	

SD= standard deviation; BP= blood pressure; bpm= beat per minute; LVEF= left ventricular ejection fraction; PWD= P wave dispersion; MR= mitral regurgitation; TR= tricuspid regurgitation; AR= aortic regurgitation; AS= aortic stenosis; PAP= pulmonary artery pressure

**Table 3:** Electrocardiographic or echocardiographic changes according to PASI score

**Variable**	**PASI**			**P value**
	**<7**	**7-12**	**>12**	
P max, msec (mean±SD)	79.71±14.92	77.12±7.56	60±0.01	0.369
P min, msec (mean±SD)	38.57±7.32	37.14±7.32	36±0.01	0.847
PWD, msec (mean±SD)	41.50±11.63	40±6.16	24±0.02	0.286
Diastolic dysfunction number (percentage)	10 (35.7)	5 (71.4)	1 (100)	0.142
Increased PAP number (percentage)	12 (42.9)	2 (28.6)	0 (0)	0.802

PASI= Psoriasis Area Severity Index; SD= standard deviation; PAP= pulmonary artery pressure

## Discussion

Our findings primarily suggest that patients with psoriasis have higher PWD, which make them susceptible to develop arrhythmias such as AF. Various studies have already demonstrated the relationship between psoriasis and cardiovascular events (15-17). Aortic regurgitation, mitral valve prolapse, dilated cardiomyopathy, Aortitis, sudden cardiac death and acute myocardial infarction have all been reported in psoriasis patients (18). Although Markuszeski et al. claimed in a 2007 study with a low sample size that the heart rate increased in psoriasis patients (19), our study demonstrated no association between psoriasis and heart rate, left ventricular ejection fraction, valvular heart diseases or diastolic function a result that was similar to a 2015 study conducted by Metta et al. (20).

In our study, older psoriasis patients (aged 60 and higher) all had grade I left ventricular diastolic dysfunction which was significant compared with other age ranges. This finding might be due to their age exclusively and not related to psoriasis itself; as a considerable portion of older patients in the control group also had left ventricular diastolic dysfunction. This is similar to a study done by Tsigaridas et al, who showed there is no significant increase in number of patients with diastolic dysfunction in psoriasis group (13). 

There was no significant difference between the ejection fractions of the two groups which is in line with results from the study done by Tsigaridas et al. and against the study done by Skokr et al. (13, 14), who showed significant difference between the ejection fractions of the two groups. Although statistically significant, the difference noted was not clinically significant. Both these studies also evaluated subclinical decline of left ventricular (LV) function and showed impaired global longitudinal strain in the psoriasis group (13, 14). 

We also found that there were more number of patients having pulmonary artery hypertension (pressure>30 mmHg) significantly in the psoriasis group. However, Poorzand et al. published contrary results, reporting all psoriatic patients to have a normal pulmonary artery pressure of 25-30 mmHg (1) a finding that may have been by due to their lower sample size (23 patients and 23 healthy controls). Our findings were similar to Gunes et al. in 2008 study, who found more number of abnormal PAP in patients with psoriasis (21). However, they state that this may be due to the higher BMI which may be a risk factor for both psoriasis and higher PAP. In our study both groups had similar BMI and the difference in PAP between the groups can be attributed to the inflammatory mechanisms underlying this disease which can affect the vascular system. In our study, PWD on the standard 12-lead electrocardiogram was significantly higher in psoriatic patients than the control group. This finding is similar to some other studies (9, 17, 20). Metta et al. also reported a decreased P min in psoriasis group that we similarly observed in our study. P max however, was significantly higher in psoriatic patients of their study, while we did not get such a result in ours. This may be due to the fact that these patients had lower P min values rather than having a high P max.

Furthermore, we attempted to evaluate the relationship between p-wave changes and the severity of psoriasis based on PASI; however, because our sample included few psoriasis patients with moderate and severe PASI’s (most of the 36 psoriasis patients who entered our study, had the mild form of the disease (PASI < 7), while only one of them had the severe form (PASI > 12), we could not perform a reliable analysis. This was also the case when we decided to assess the relationship between the duration of the disease and PASI. Another limitation that we faced was not knowing the baseline characteristics in terms of known risk factors for arrhythmias such as hypertension and coronary artery disease. These factors could have had a confounding effect in our results if they were not similar in both groups. The sample size was also based on changes of PWD which may have resulted in not finding significant differences between the LV function of the two groups. We suggest future studies be conducted with greater sample sizes and sufficient amounts of patients from each PASI group (low, moderate and severe). 

In conclusion based on our findings, psoriatic patients have more PWD and therefore are susceptible to future development of atrial fibrillation because of higher PWD. There is no significant difference between the diastolic function in these patients. Also, increased pulmonary artery pressure is more common in psoriatic patients than the normal population. The authors suggest all newly diagnosed psoriatic patients be evaluated for cardiovascular function. We also recommend a regular cardiovascular follow-up schedule to be considered for these patients. 
